# Fibrosing mediastinitis complicating prior histoplasmosis is associated with human leukocyte antigen DQB1*04:02 − a case control study

**DOI:** 10.1186/s12879-015-0943-7

**Published:** 2015-05-05

**Authors:** Stephen B Strock, Silvana Gaudieri, Simon Mallal, Chang Yu, Daphne Mitchell, Joy Cogan, Wendi Mason, Deborah Crowe, James E Loyd

**Affiliations:** Department of Medicine, Vanderbilt University, 1211 Medical Center Dr, Nashville, TN 37232 USA; School of Anatomy, Physiology and Human Biology, University of Western Australia, 35 Stirling Highway, Crawley, WA 6009 Australia; Institute for Immunology & Infectious Disease, Murdoch University, Health Research Centre, Discovery Way, Murdoch, WA 6150 Australia; Department of Infectious Disease and Pathology, Microbiology and Immunology, Vanderbilt University, 1211 Medical Center Dr, Nashville, TN 37232 USA; Department of Biostatistics, Vanderbilt University, 1211 Medical Center Dr, Nashville, TN 37232 USA; Pulmonary and Critical Care Medicine, Vanderbilt University, 1211 Medical Center Dr, Nashville, TN 37232 USA; Division of Medical Genetics and Genomic Medicine, Department of Pediatrics, Vanderbilt University, 1211 Medical Center Dr, Nashville, TN 37232 USA; DCI Laboratory – Transplant Immunology, 1616 Hayes St, Nashville, TN 37203 USA

**Keywords:** *Histoplasma capsulatum*, Fibrosing mediastinitis, Human leukocyte antigen

## Abstract

**Background:**

Fibrosing mediastinitis (FM) is an idiosyncratic reaction to infection with *Histoplasma capsulatum* with a prevalence of 3:100,000 people infected. The rarity of post-histoplasmosis fibrosing mediastinitis (PHFM) in areas where *H. capsulatum* is endemic suggests that an abnormal immunological host response may be responsible for the development of fibrosis. Our group previously reported an association between subjects with PHFM and human leukocyte antigen (HLA)-A*02. We sought to confirm or extend those findings with application of high resolution HLA typing in a cohort of subjects with PHFM.

**Methods:**

High-resolution HLA typing was performed on DNA samples from a new cohort 34 patients with PHFM. Control cohorts included 707 subjects from the “European American” subset of the National Marrow Donor Program® (NMDP) and 700 subjects from Dialysis Clinic, Inc. (DCI). The carriage frequencies of the HLA alleles identified in the PHFM, NMDP, and DCI cohorts were calculated and then all were compared.

**Results:**

We found an increase in the carriage frequency of HLA-DQB1*04:02 in PHFM subjects relative to the controls (0.15 versus 0.07 in DCI and 0.05 in NMDP; p = 0.08 and 0.03). Multiple logistic regression showed that DQB1*04:02 was statistically significant (p = 0.04), while DQB1*03:02 and C*03:04 had point estimates of OR > 1, though they did not reach statistical significance. The HLA-A*02 association was not replicated.

**Conclusions:**

HLA-DQB1*04:02 is associated with PHFM, which supports the premise that an aberrant host immune response contributes to the development of PHFM.

## Background

Fibrosing mediastinitis (FM) is characterized by excessive proliferation of invasive fibrous tissue within the mediastinum. In North America the vast majority of cases of FM result from an idiosyncratic reaction to infection with *H. capsulatum*, a dimorphic fungus endemic to the Mississippi and Ohio River valleys in the United States, as well as temperate zones worldwide [1-4]. Other causes of FM are seen very rarely, including an idiopathic form sometimes associated with retroperitoneal fibrosis, IgG4-related disease, or medication (methysergide) toxicity [1-3,5].

Infection with *H. capsulatum* is usually asymptomatic and inconsequential, but in some individuals acute histoplasmosis may cause malaise, fever, and cough. A few patients with acute histoplasmosis develop symptomatic mediastinal adenitis, often in the subcarinal or right paratracheal area, with a characteristic central chest pain worse during inspiration. Acute mediastinal adenitis is the origin of subsequent unique mediastinal complications, including mediastinal granuloma and FM. Mediastinal granuloma describes a mass, often large (>4 cm), of coalescent lymph nodes in which a thin capsule contains semiliquid contents, and is usually asymptomatic.

In contrast, post-histoplasmosis fibrosing mediastinitis (PHFM) is solid, with dense and invasive fibrosis. The fibrous proliferative mass contains ectopic calcification on CT scan, and is dense, typically described by surgeons as “concrete,” arising from encircling lymph nodes that were the site of remote mediastinal adenitis. Its clinical consequences are due to invasion of normal mediastinal structures such as the pulmonary vasculature, the superior vena cava, or the airways in any combination [1-3,6]. A typical presentation is auto-amputation of the right pulmonary artery; obstruction of vessels of both lungs is less common, but the mortality is high. The diagnosis of PHFM is based on clinical and radiographic findings as above, as well as the exclusion of malignancy, radiation therapy, or thromboembolic disease [1]. This rare complication of histoplasmosis has an observed prevalence of 3 per 100,000 cases [7]. Although nearly everyone who lives in an endemic region is infected with *H. capsulatum* intermittently, it is unclear why some individuals rarely develop PHFM, and suggests a possible abnormal immunological host response to infection.

The genes encoding the HLA system are located on chromosome 6. The HLA class I (HLA-A, −B, −C) molecules are present on most somatic cells, whereas the HLA class II (HLA-DM, −DO, −DP, −DQ, −DR) molecules are typically expressed by antigen presenting cells. Both classes are important for antigen processing and presentation as part of the adaptive immune system [8,9].

Our group previously reported an association between subjects with PHFM and the HLA class I molecule, HLA-A*02. We sought to confirm or extend those findings with application of high resolution HLA typing in a cohort of subjects with PHFM, as high resolution typing may be more useful than low resolution typing for the determination of likely causative genes rather than linked variations [10]. There are differences in peptide specificity of HLA subtypes, which potentially correspond to different disease phenotypes [11,12]. Our hypothesis is that an intrinsic inherited characteristic, represented by HLA type, predisposes individuals to excessive host reaction to histoplasmosis, manifested clinically as PHFM.

## Methods

### Case subjects

We recruited 34 consecutive patients with a diagnosis of PHFM who presented to the Vanderbilt University Medical Center between 2011 and 2013. PHFM was defined clinically as excessive proliferation of fibrous tissue within the mediastinum causing invasion of normal mediastinal structures. The typical calcifications seen within the invasive fibrous tissue on chest radiography helped delineate PHFM from other mediastinal abnormalities [3]. All patients had serology or imaging that confirmed prior histoplasmosis [1]. Patients with a history of mediastinal radiation therapy or malignancy involving the mediastinum were excluded. None of the patients in this study had increased serum IgG4 levels, although it was not clinically tested in all patients [5]. Given the genetic diversity seen between different ethnic groups, we selected only case subjects of European ancestry as determined by self-report. Other covariates were also collected, including: age, gender, geographic location, anatomic structures involved, clinical outcome, and comorbid conditions.

### Population control subjects

Population controls were obtained from the NMDP, which has made high-resolution haplotype frequencies from 21 detailed population categories available (http://bioinformatics.nmdp.org). For our population control, we used the data from only the “European American” subset from 2012-present, consisting of 707 subjects. HLA carriage frequencies were available at the group level, but not at the individual level.

Another set of population controls was obtained from DCI Laboratory in Nashville, TN, which is the local HLA laboratory for transplant programs. We queried for results from 2012 – present, resulting in 700 cases. Ethnicity of these controls is not available, but HLA carriage frequencies were available at the individual level. We did not screen either of our population controls for evidence of histoplasmosis or PHFM.

### HLA genotyping of PHFM patients

High-resolution four-digit typing of the HLA Class I (HLA-A, −B, −C) and HLA Class II (HLA-DRB1, DQB1, DQA1, DP, DR3, DR4 and DR5) loci was performed on DNA samples using the 454 Roche DNA pyrosequencer with GS FLX chemistry (Roche 454 FLX technology) utilizing liquid handling robotics and a local database tracking system. Briefly, multiplex identifier primers targeting exons 2 and 3, containing the peptide determining regions, were used to amplify each locus separately and then pooled. HLA allele genotypes were analyzed for each individual and assigned based on the HLA allele sequences in the immunogenetics information system database (http://www.imgt.org).

### Statistical methods

This is a case–control study. Standard epidemiological methods for analyzing case–control data were utilized [13]. For each allele, the carriage frequencies were compared between FM cases and controls using a chi-squared test. Multiple logistic regressions were used to analyze multiple loci simultaneously and this is the primary analysis [13,14]. However, with a total of 34 FM cases, the model is limited to be able to include only three loci at a time without over fitting.

## Results

Of the 34 patients, 21 were male and 13 were female. All were Caucasian. The mean age (± SEM) at the time of diagnosis of PHFM was 41.2 ± 10.1 years. Table 1 shows the summary data for the group.

**Table 1:** **Baseline characteristics of the study patients**

Gender	Male: 21 patients (61%)	Female: 13 patients (39%)
Age	41.2 ± 10.1 years	
Geographic location	AL, CA, GA, IA, IL, IN, KY, MI, MO, MS, OH, TN, TX, VA	
Anatomic site involved	Unilateral: 18 (53%)	Bilateral: 16 (47%)

The carriage frequencies of the HLA alleles in the PHFM cohort were calculated and compared to corresponding frequencies determined in the DCI and the NMDP control cohorts. We found a significant increase in the carriage frequency of HLA-DQB1*03:02 in the PHFM cohort relative to controls (0.32 versus 0.18 in both control cohorts; p = 0.04). Similarly, there was an increase in the frequency of HLA-DQB1*04:02 in subjects with PHFM relative to the control cohorts (0.15 versus 0.07 in DCI controls and 0.05 in NMDP controls; p = 0.08 and 0.03). There was a borderline association between HLA-C*03:04 and PHFM (carriage frequency of 0.29 versus 0.16 in both control cohorts; p = 0.06 and 0.05). The HLA-A*02 association from the 2000 study was not replicated, as there was no significant difference in the carrier frequency compared to both control cohorts (0.50 versus 0.47 in DCI controls and 0.50 in NMDP controls) [4].

A multiple logistic regression with HLA-C*03:04, HLA-DQB1*03:02, and HLA-DQB1*04:02 in the model was conducted. The odds ratios for these factors are reported in Table 2. Among the three factors, HLA-DQB1*04:02 was statistically significant (p = 0.044) and the other two had a point estimate of OR greater than 1, though they did not reach statistical significance.

**Table 2:** Logistic regression with C*03:04, DQB1*03:02, DQB1*04:02 in the model

	**Odds ratio estimates**			
**Effect**	**Point estimate**	**95% Wald confidence limits**		**p-value**
C*03:04	1.670	0.718	3.886	0.23
DQB1*03:02	1.971	0.860	4.514	0.11
DQB1*04:02	2.836	1.030	7.809	0.044

The HLA-DQB1*03:02 allele is in strong linkage disequilibrium with HLA-DRB1*04:01, but in this study there was no association between PHFM and HLA-DRB1*04:01 (carriage frequency of 0.23 versus 0.15 in DCI controls and 0.17 in NMDP controls; p = 0.24 and 0.37). The HLA-C*03:04 is not in strong linkage disequilibrium with either of the two HLA-DQB1 alleles increased in the PHFM cohort in Caucasian populations (www.allelefrequencies.net) and may reflect different effects. However, in the 10 individuals carrying the HLA-C*03:04 allele, six also carried HLA-DQB1*03:02 and of these six individuals four also carried HLA-B*15:01, which is an extended haplotype combination observed at low frequency in some European populations (www.allelefrequencies.net). In this study, HLA-B*15:01 is not associated with PHFM (carriage frequency of 0.14 versus 0.15 in DCI controls and 0.13 in NMDP controls; p = 1.00 and p = 0.80).

## Discussion

FM is the most serious late complication of infection with *H. capsulatum*. Only a small minority of all individuals infected with *H. capsulatum* go on to develop PHFM, so it has long been suspected that these patients have some unique predisposition. Our hypothesis is that an intrinsic inherited characteristic, related to HLA type, predisposes individuals to excessive host reaction to histoplasmosis, manifested clinically as PHFM.

As has now been established for many autoimmune and infectious diseases, genetic variation at the HLA loci is a primary determinant of genetic risk – an observation that has persisted into the era of genome-wide association studies. Examples of diseases that have been associated with HLA molecules include: ankylosing spondylitis with HLA-B*27; type 1 autoimmune hepatitis with HLA-DRB1*03:01 and HLA-DRB1*04:01; insulin-dependent type 1 diabetes mellitis and celiac disease with HLA-DQB1*03:02; primary biliary cirrhosis, neuromyelitis optica, and cervical cancer with HLA-DQB1*04:02; and anti-citrullinated antibody positive rheumatoid arthritis with HLA-C*03:04 [15-21]. In this investigation we describe the first report of an association between the HLA Class II molecule, HLA-DQB1*04:02, and patients with PHFM. We also found a trend toward significance with HLA-DQB1*03:02 and HLA-C*03:04. The HLA-A*02 association from the 2000 study was not replicated. When that study was performed, antibody-based serotyping was used in which antibodies bind to the exposed extra-cellular aspects of an HLA molecule [4]. This methodology can only define a group of HLA alleles, but not unique amino acid sequences, as can be delineated by the PCR-based methods used in this study. Since the advent of high-resolution four-digit HLA genotyping, HLA-A*02 has been found to be a commonly represented allele group (www.allelefrequencies.net), making the results of the 2000 study likely a result of chance.

Multiple mechanisms have been proposed to explain the association between HLA molecules and disease susceptibility. Peptide motifs fit specifically into complementary pockets on the HLA binding sites, thus determining which peptides will be presented to immune cells. A recent study showed that abacavir binds inside the antigen-binding site of HLA-B*57:01, allowing the presentation of peptides that normally would not be able to bind, which could be perceived as foreign by T-cells and trigger a response [12]. During T-cell development, antigen presentation by HLA molecules helps with positive and negative selection, thus determining which T-cells will survive and move into the periphery, including possibly auto-reactive T-cells. Molecular mimicry between the epitope of the bound peptide molecule and self-antigens could additionally lead to auto-reactive T-cells or reactivation of silenced T-cells [22]. In a recent study, Raychaudhuri et al. were able to show that three specific single amino acid polymorphisms located within the peptide-binding groove of HLA-DRB1 help explain the association between the HLA allele and risk of anti-CCP rheumatoid arthritis [23]. Future work on the association between HLA-DBQ1*04:02 and risk of PHFM could attempt to identify similar amino acid polymorphisms with the peptide binding groove of the HLA Class II molecule, and eventually help identify a specific pathogenic auto-antigen that may lead to PHFM.

Other investigators recently reported immunophenotyping of mixed inflammatory cells infiltrating the mediastinal tissues of subjects with PHFM. They identified an abundance of CD20^+^ B lymphocytes, which suggests their possible immunopathogenic role [2]. This finding is consistent with an HLA Class II molecule like HLA-DBQ1*04:02 prompting a response from its presented antigen by CD4+ Th2 helper cells. Th2 cytokines, such as IL-4, IL-5, IL-13, and IL-21, provide paracrine signals that activate myofibroblasts. B-cell activation stimulates production of pro-fibrotic cytokines like IL-6 and possibly autoantibodies. Activated macrophages and monocytes produce TGF-β, which can directly activate mesenchymal cells to transform into myofibroblasts and upregulate transcription of genes like procollagen I & III via Smad protein intermediates [24].

At this point therapeutic options for patients with PHFM are extremely limited. Neither antifungal therapy directed at *H capsulatum* nor anti-inflammatory therapy with glucocorticoids have proven beneficial, although B lymphocyte depletion with an agent such as rituximab has been suggested as an alternative [1,2]. It is our hope that further elucidation of the etiology underlying the idiosyncratic inflammatory reaction believed to cause PHFM may eventually lead to the development of novel therapeutic approaches to patients with this condition.

### Limitations

This study was limited by small sample size since PHFM is a rare disease. With a larger sample, it is possible that the associations seen between PHFM and both HLA-DQB1*03:02 and HLA-C*03:04 would become statistically significant. Additionally, past HLA association studies have shown that ethnicity is an important part of the risk associated with HLA type for a specific disease. The ideal control group would be a large, regional Caucasian database with HLA carriage frequencies available at the individual level for statistical analysis. The NMDP control cohort was restricted to those of “European American” ancestry; however, carriage frequencies were only available for the group as a whole. On the other hand, the DCI control cohort was from Nashville and had individual carriage frequencies available, but ethnicity was not reported. Since the carriage frequencies between the two control cohorts were not statistically different, this suggests that the lack of ethnicity data from the DCI cohort did not affect the results.

## Conclusions

PHFM patients exhibit excessive fibrotic proliferation that can auto-amputate major vessels and airways. In this investigation we describe the first report of an association between HLA-DQB1*04:02 and patients with PHFM. An HLA Class II association supports the premise that PHFM patients carry an underlying idiosyncratic predisposition that drives excessive fibrous proliferation when provoked by exposure to *H. capsulatum* organisms.

## Abbreviations

DCI: Dialysis Clinic, Inc.
FM: Fibrosing mediastinitis
HLA: Human leukocyte antigen
MHC: Major histocompatibility complex
NMDP: National Marrow Donor Program
PHFM: Post-histoplasmosis fibrosing mediastinitis
